# Supplementary material for: HIVprotI: an integrated web based platform for prediction and design of HIV proteins inhibitors
Source: J Cheminform. 2018 Mar 9;10:12. doi: 10.1186/s13321-018-0266-y (PMC5845081; doi:10.1186/s13321-018-0266-y)
Supplement: Supplementary file 2 — Additional file 2. Source code of HIVProtI web server. [file 13321_2018_266_MOESM2_ESM.zip › HIVprotI_Source-code/hivprotI/weka-3-6-6/doc/weka/associations/tertius/package-summary.html]

weka.associations.tertius


---


|  |  |  |
| --- | --- | --- |
| **Overview** \| **Package** \| Class \| **Tree** \| **Deprecated** \| **Index** \| **Help** \| **Weka's home** | |  |
| **PREV PACKAGE**   **NEXT PACKAGE** | **FRAMES**    **NO FRAMES**     **All Classes** |  |


---

## Package weka.associations.tertius

| **Class Summary** | |
| --- | --- |
| **AttributeValueLiteral** |  |
| **Body** | Class representing the body of a rule. |
| **Head** | Class representing the head of a rule. |
| **IndividualInstance** |  |
| **IndividualInstances** |  |
| **IndividualLiteral** |  |
| **Literal** |  |
| **LiteralSet** | Class representing a set of literals, being either the body or the head of a rule. |
| **Predicate** |  |
| **Rule** | Class representing a rule with a body and a head. |
| **SimpleLinkedList** |  |

---


|  |  |  |
| --- | --- | --- |
| **Overview** \| **Package** \| Class \| **Tree** \| **Deprecated** \| **Index** \| **Help** \| **Weka's home** | |  |
| **PREV PACKAGE**   **NEXT PACKAGE** | **FRAMES**    **NO FRAMES**     **All Classes** |  |


---
